# État nutritionnel des enfants traités pour paludisme grave en milieu hospitalier dans le Haut-Katanga (République démocratique du Congo)

**DOI:** 10.11604/pamj.2026.53.136.48719

**Published:** 2026-03-23

**Authors:** Gray Kanteng, Cédric Mwepu, Augustin Mutombo, Numbi Luboya

**Affiliations:** 1 Département de Pédiatrie, Faculté de Médecine, Université de Lubumbashi, Lubumbashi, République démocratique du Congo

**Keywords:** Paludisme, malnutrition, enfant, Malaria, malnutrition, child

## Abstract

La présente étude a porté sur l'état nutritionnel des enfants de moins de 5 ans admis et suivis pour paludisme en milieu hospitalier dans les villes de Lubumbashi et Likasi. L'objectif était de dresser le profil nutritionnel de ces enfants, basé sur l'anthropométrie. Il s'agit d'une étude descriptive transversale effectuée dans la province du Haut-Katanga, au sud-est de la République démocratique du Congo. Ont été inclus les enfants de moins de 60 mois, dont le diagnostic positif est celui du paludisme, confirmé biologiquement par un TDR positif et/ou une goutte épaisse positive. L'incidence du paludisme était de 34,3%. La prévalence globale de la malnutrition aiguë (modérée à sévère) est de 32,9%, représentée essentiellement par la malnutrition sévère avec 27,5%. La malnutrition aiguë est plus prévalente dans sa forme modérée (67,1%), mais présente dans quasiment toutes les catégories d'âge. Le surpoids a été rencontré chez 13,9% d'enfants; il était sévère chez 8,5% d'enfants. Une association systématiquement significative a été observée entre l'insuffisance pondérale modérée à sévère (Z-score <-2 ET), le retard de croissance modéré à sévère (Z-score <-2 ET), la malnutrition aiguë modérée à sévère (Z-score <-2 ET) et le paludisme simple plutôt qu'avec le paludisme grave. Il ressort une fréquence relativement élevée des cas d'insuffisance pondérale, d'émaciation et de retard de croissance, souvent plus importante que celle documentée dans la population générale. Des études d'impact sont nécessaires pour établir un éventuel lien de causalité.

## Abstract (en)

This study assessed the nutritional status of children under five years of age admitted and treated for malaria in hospital settings in the cities of Lubumbashi and Likasi. The purpose of this study was to describe the nutritional profile of these children based on anthropometric measurements. We conducted a cross-sectional descriptive study conducted in the Haut-Katanga province in southeastern Democratic Republic of the Congo. Children under 60 months of age with a positive diagnosis of malaria, biologically confirmed by a positive RDT and/or a positive thick blood smear, were included. The incidence of malaria was 34.3%. The overall prevalence of acute malnutrition (moderate to severe) was 32.9%, predominantly represented by severe acute malnutrition (27.5%). Acute malnutrition was more frequent in its moderate form (67.1%) and was observed across nearly all age groups. Overweight was identified in 13.9% of children, including 8.5% with severe overweight. A consistently significant association was observed. This study focused on the nutritional status of children under 5 years of age admitted and monitored for malaria in hospital settings in the cities of Lubumbashi and Likasi. The objective was to outline the nutritional profile of these children based on anthropometric measurements. This was a descriptive cross-sectional study conducted in the Haut-Katanga province, in the southeast of the Democratic Republic of Congo. Included were children under 60 months of age with a positive diagnosis of malaria, biologically confirmed by a positive rapid diagnostic test (RDT) and/or a positive thick blood smear. The incidence of malaria was 34.3%. The overall prevalence of acute malnutrition (moderate to severe) was 32.9%, mainly represented by severe malnutrition at 27.5%. Acute malnutrition was more prevalent in its moderate form (67.1%), but was present in almost all age categories. Overweight was observed in 13.9% of children, with severe overweight in 8.5% of children. A consistently significant association was observed between moderate to severe underweight (Z-score <-2 SD), moderate to severe stunting (Z-score <-2 SD), moderate to severe acute malnutrition (Z-score <-2 SD), and uncomplicated malaria rather than severe malaria. There is a relatively high frequency of cases of underweight, wasting, and stunting, often higher than that documented in the general population. Impact studies are needed to establish a potential causal link.

## Introduction

La sous-alimentation est l'une des deux principales causes de mortalité de l'enfant, notamment en Afrique [1]. Les pays les plus touchés par la malnutrition sont généralement les mêmes que ceux où le paludisme cause une importante morbidité [2]. Alors que la malnutrition par excès, en l'occurrence le surpoids et l'obésité, devient un fléau mondial, la malnutrition carentielle continue à causer des ravages dans la population infantile. En 2019 et en 2020, cette part était respectivement de 8% et de 9,3%. En 2021, quelque 2,3 milliards de personnes (29,3% de la population mondiale) étaient en situation d'insécurité alimentaire modérée ou grave - soit 350 millions de personnes de plus qu'avant la pandémie de COVID-19 [3]. L'Afrique subsaharienne présente certains des taux les plus élevés au monde de malnutrition chez l'enfant (39% du retard de croissance et 10% de la malnutrition infantile sur le classement mondial) selon l'Organisation mondiale de la Santé (OMS) [4].

La situation de la malnutrition en République démocratique du Congo (RDC) demeure préoccupante depuis les dernières décennies. La RDC est un des dix pays qui représentent 60% de la charge mondiale de l'émaciation chez les enfants de moins de 5 ans. En 2022, on estime à 2,8 millions le nombre de personnes souffrant de malnutrition aiguë globale, dont 1,2 million d'enfants de moins de cinq ans [5]. De l'autre côté, en RDC, les chiffres montrent que la malaria demeure un des problèmes de santé publique majeurs. En 2016, 13,4% des décès d'enfants de moins de cinq ans étaient dus au paludisme [6]. Cette même année, environ 47% des épisodes de paludisme sont survenus chez des enfants de moins de cinq ans [7]. Le paludisme grave est responsable de 77% des hospitalisations d'enfants de moins de cinq ans et de 55% des hospitalisations de patients de plus de cinq ans [8].

Il ressort donc qu'ensemble combinés, le paludisme et la malnutrition représentent un fardeau important qui préjudicie la survie de l'enfant congolais [9,10]. Pourtant, il existe un déficit d'information quant à ce fléau combiné, et quand les approches cliniques ou de prise en charge sont faites, elles concernent souvent soit la malnutrition, soit le paludisme.

Ce travail a pour but de combler ce déficit. Il vise à décrire l'état nutritionnel des enfants de moins de 5 ans atteints de paludisme et à évaluer l'influence de l'état nutritionnel sur le faciès clinique et évolutif du paludisme. L'objectif général est d'évaluer l'état nutritionnel des enfants âgés de 0 à 59 mois souffrant de paludisme en milieu hospitalier d'endémie dans la région du Haut-Katanga (RD Congo). Les objectifs spécifiques sont de déterminer l'incidence du paludisme chez les enfants âgés de 0 à 59 mois et de décrire le statut nutritionnel des enfants atteints de paludisme.

## Méthodes

**Description du cadre d'étude:** la présente étude a été menée en milieu hospitalier, dans 3 structures urbaines situées dans la région du Haut-Katanga (RD Congo). Il s'agit de l'hôpital général Jason Sendwe (Lubumbashi), des cliniques universitaires de Lubumbashi (Lubumbashi) et de l'hôpital du personnel Gécamines Panda (Likasi). Le Haut-Katanga est une province de la République démocratique du Congo, située au Sud-Est. Lubumbashi est le chef-lieu et Likasi, la deuxième ville de la province en termes d'importance démographique et d'infrastructures. Les deux villes sont séparées par une distance de 120 km. La région a un climat tropical, marqué par deux saisons: une saison sèche qui va de mai à octobre, et une saison des pluies qui va de novembre à avril. Les températures moyennes varient dans l'année de 10°C à 40°C [11].

**Population, durée et type d'étude:** il s'agit d'une étude descriptive et transversale, effectuée sur un mode multicentrique. La période qu'embrasse l'étude est de 5 mois, soit de septembre 2021 à janvier 2022.

**Type d'échantillonnage:** un échantillonnage exhaustif de cas a été colligé, selon les critères d'inclusion décrits plus bas.

**Taille de l'échantillon:** pour le calcul de la taille de l'échantillon minimum, elle s'est faite suivant la formule ci-après: n= (e^2^•p•q)/d^2^. Pour un échantillon minimum attendu de n + n/10. Avec: i) n: l'échantillon minimum; ii) e: constante = 1,96; iii) p: prévalence de la pathologie ou probabilité de l'événement étudié; iv) q: le complément de p = 1-p et v) d: l^er^ degré de précision = 5%. Dans la présente étude, « l'événement » correspond à la prévalence du paludisme chez l'enfant en milieu hospitalier. Une étude antérieure menée à l'état de la RD Congo, a trouvé une prévalence de 35,8% [1] de paludisme chez l'enfant en milieu hospitalier; valeur de calcul de base dans notre étude. Ainsi calculé, n = 353,1; soit un échantillon minimal attendu de 354 (n) + 36 (n/10) = 390 cas. Nous avons pour notre part colligé 400 cas, à partir desquels la collecte de données a été interrompue.

**Critères d'inclusion:** les critères d'inclusion dans l'étude ont été les suivants: enfant de moins de 60 mois, dont le diagnostic positif est celui du paludisme, confirmé biologiquement par un TDR positif et/ou une goutte épaisse positive.

**Critères de non-inclusion:** les critères de non-inclusion ont été les suivants: i) les enfants présentant une pathologie associée au paludisme; ii) les patients suspects de paludisme dont la confirmation du diagnostic n'a pas été faite.

**Technique de collecte des données:** voici les différents matériels qui ont permis la collecte des données: i) une fiche de collecte contenant un questionnaire rempli après l'examen physique des nourrissons et par interview des accompagnateurs; ii) une toise, pour la mesure de la taille, prise en position couchée, par le concours de 2 personnes, l'occiput, les omoplates, les fesses et les jambes du nourrisson bien à plat sur le plan horizontal. La longueur est notée en cm; iii) un mètre ruban, pour la mesure du périmètre brachial, au niveau du bras gauche, à mi-distance entre l'olécrane et l'acromion. Il a également servi pour la mesure du périmètre crânien; iv) une balance SALTER, pour la mesure du poids, exprimé en kg.

**Analyses statistiques:** la détermination de l'état nutritionnel s'est faite sur la base des indicateurs suivants: poids, taille et âge, permettant le calcul du z-score. Ce dernier a été calculé à l'aide du logiciel Ena version 2007 (*Emergency Nutrition Assessment*) et l'état nutritionnel a été classifié de la manière suivante: état nutritionnel normal (Z-score supérieur ou égal à -1,00), malnutrition légère (Z-score compris entre -2,00 et -1,01), malnutrition modérée (Z-score compris entre -3,00 et -2,01) et malnutrition sévère (Z-score inférieur à -3,00) [2]. Les courbes de croissance de référence utilisées étaient celles de l'OMS (2006). Les valeurs aberrantes dans les données anthropométriques ont été exclues de l'analyse. Les limites d'exclusion ont été définies à ±- 3 DS du Z-score PPT par rapport à la moyenne de PPT observée; ainsi, 11 résultats Z-score PPT ont été exclus de l'analyse. L'encodage des données a été fait à l'aide du logiciel Microsoft Excel 2013. Le traitement des données a été effectué à l'aide du logiciel Epi Info version 7 (2013). Les tests statistiques nécessaires ont été calculés, notamment la moyenne et l'écart-type, la médiane, les quartiles. Lorsqu'une association est effectuée entre deux ou plusieurs paramètres, le rapport des cotes (odds ratio) a été calculé et/ou le test du khi carré (de Yates ou de Fisher lorsque recommandé) avec un intervalle de confiance à 95%. Le seuil de signification est fixé à p < 5%. Les données sont étudiées en deux temps : une analyse univariée pour la description des principaux paramètres étudiés, et une analyse multivariée pour déterminer les relations d'association entre ces paramètres.

**Considérations éthiques:** l'étude a été effectuée sous le consentement verbal libre et éclairé des parents présents de chaque enfant, après une explication brève du but de notre étude. Le respect de la confidentialité des sujets de notre étude a été assuré. Le protocole du présent travail a été soumis à l'approbation au Département de Pédiatrie de la Faculté de Médecine de l'Université de Lubumbashi. Il a également été accepté par le comité d'éthique de l'université de Lubumbashi.

## Résultats

**Figure 1:**
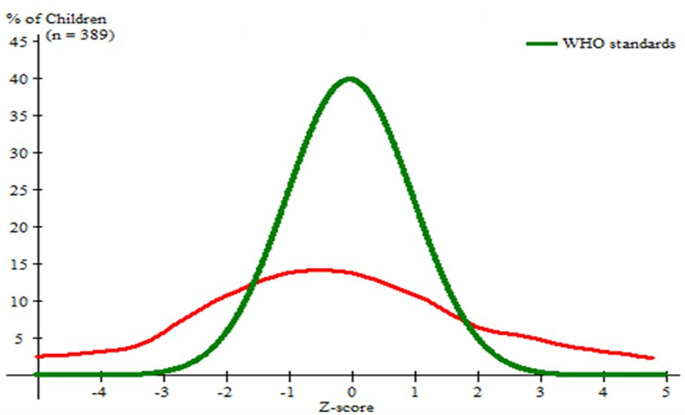
distribution de l'échantillon selon le Z-score poids pour âge (*weight-for-height z-scores*)

L'incidence du paludisme était de 34,3% (Tableau 1). Les cas ont été colligés de manière équilibrée dans les villes de Likasi et de Lubumbashi. La majorité des mères ont déclaré être employées dans le secteur public ou privé (64,3%). Le sexe ratio garçons/filles est de 0,8. La majorité des cas était retrouvée avant 30 mois, autant chez les filles que chez les garçons (Tableau 2). Il ressort de la Figure 1 que la courbe de la population d'étude s'étale sous la courbe de référence, avec des déviations vers les extrêmes à gauche et à droite, soit une représentation sensible de la population pour ce qui est de la proportion attendue de l'insuffisance staturopondérale ou de l'excès staturopondéral. La prévalence globale de la malnutrition aiguë (modérée à sévère) est de 32,9%, représentée essentiellement par la malnutrition sévère avec 27,5% (Tableau 3).

**Tableau 1:** caractéristiques générales de l'échantillon

Paramètres		Effectif	Pourcentage
Nombre de cas de paludisme		400	34,3
Autres cas		1164	65,7
Provenance	Likasi	187	46,8
	Lubumbashi	213	53,2
Profession des mères	Femme au foyer	99	24,7
	Profession libérale	44	11,0
	Employée du secteur public ou privé	257	64,3

**Tableau 2:** répartition de l'âge et du sexe de l'échantillon

Sexe	Masculin		Féminin		Total	
**Âge (mois)**	**N**.	**%**	**N**.	**%**	**N**.	**%**
**6-17**	54	33,3	108	66,7	162	40,5
**18-29**	74	63,2	43	36,8	117	29,3
**30-41**	11	20,0	44	80,0	55	13,8
**42-53**	44	66,7	22	33,3	66	16,5
**54-59**	0	0	0	0	0	0,0
**Total**	183	45,8	217	54,3	400	100,0

**Tableau 3:** prévalence de la malnutrition aiguë basée sur les Z-scores du rapport poids/taille et par sexe

-	Tous n = 389	Garçons n = 172	Filles n = 217
**Prévalence de la malnutrition globale (<-2 z-score et/ou œdème)**	(128) 32,9 % (28,4 - 37,7 IC à 95 %)	(53) 30,8 % (24,4 - 38,1 IC à 95 %)	(75) 34,6 % (28,6 - 41,1 IC à 95 %)
**Prévalence de la malnutrition modérée (<-2 z-score et >=-3 z-score, pas d'œdème)**	(21) 5,4 % (3,6 - 8,1 IC à 95 %)	(0) 0,0 % (0,0 - 2,2 IC à 95 %)	(21) 9,7 % (6,4 - 14,3 IC à 95 %)
**Prévalence de la malnutrition sévère (<-3 z-score et/ou œdème)**	(107) 27,5 % (23,3 - 32,1 IC à 95 %)	(53) 30,8 % (24,4 - 38,1 IC à 95 %)	(54) 24,9 % (19,6 - 31,0 IC 95%)

**Figure 2:**
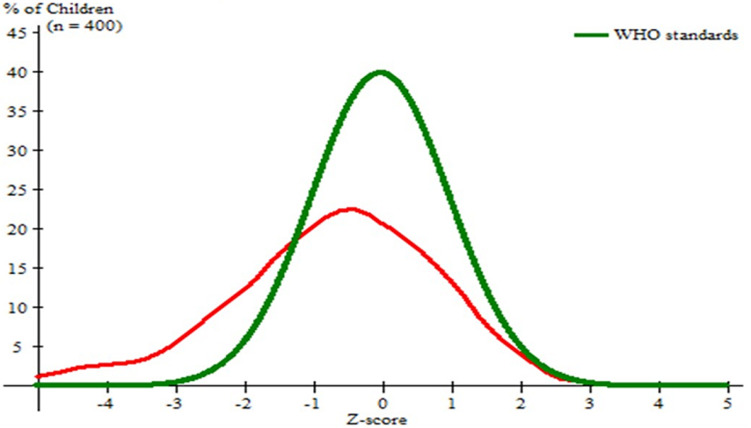
distribution de l'échantillon selon le Z-score poids pour l'âge (*weight-for-age z-scores*)

La malnutrition aiguë est plus prévalente dans sa forme modérée (67,1%), mais présente dans quasiment toutes les catégories d'âge (Tableau 4). Il ressort de la Figure 2 que la courbe de la population dévie exclusivement à gauche, ce qui représente une représentation sensible de la population pour ce qui est des valeurs attendues de l'insuffisance pondérale.

**Tableau 4:** prévalence de la malnutrition aiguë selon l'âge, sur la base des Z-scores du poids pour la taille

Paramètres		Émaciation sévère (<-3 score z)		Émaciation modérée (>= -3 et <-2 z-score )		Normal (> = -2 score z)	
**Âge (mois)**	**Nombre total**	**N**.	**%**	**N**.	**%**	**N**.	**%**
**6-17**	162	75	46,3	11	6,8	76	46,9
**18-29**	117	dix	8,5	dix	8,5	97	82,9
**30-41**	55	11	20,0	0	0,0	44	80,0
**42-53**	55	11	20,0	0	0,0	44	80,0
**54-59**	-	-	-	-	-	-	-
**Total**	389	107	27,5	21	5,4	261	67,1

**Figure 3:**
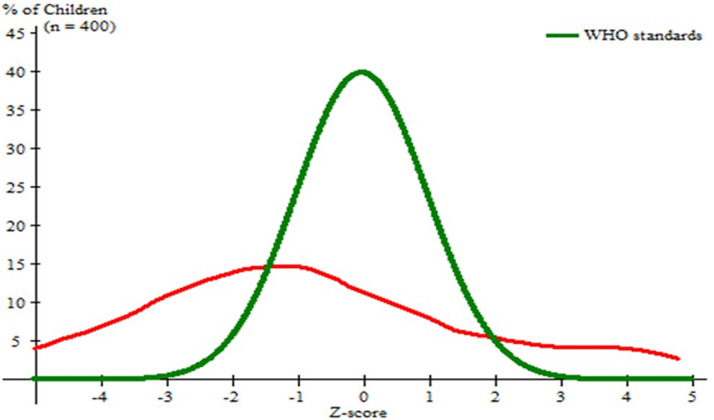
distribution de l'échantillon selon le Z-score taille pour âge

Une représentation sensible de la population est située au-dessus du seuil attendu de l'insuffisance staturopondérale. Cependant, l'échantillon est dévié vers la gauche et vers la droite (Figure 3). Le surpoids a été rencontré chez 13,9% d'enfants; il était sévère chez 8,5% d'enfants (Tableau 5). Une association systématiquement significative a été observée entre l'insuffisance pondérale modérée à sévère (Z-score <-2 ET), le retard de croissance modéré à sévère (Z-score <-2 ET), la malnutrition aiguë modérée à sévère (Z-score <-2 ET) et le paludisme simple plutôt qu'avec le paludisme grave (Tableau 6).

**Tableau 5:** prévalence du surpoids selon les seuils de poids pour la taille et selon le sexe

Paramètres	Tout n = 389	Garçons n = 172	Filles n = 217
**Prévalence du surpoids (WHZ > 2)**	(54) 13,9 % (10,8 - 17,7 IC à 95 %)	(32) 18,6 % (13,5 - 25,1 IC 95%)	(22) 10,1 % (6,8 - 14,9 IC à 95 %)
**Prévalence du surpoids sévère (WHZ > 3)**	(33) 8,5 % (6,1 - 11,7 IC à 95 %)	(11) 6,4 % (3,6 - 11,1 IC à 95 %)	(22) 10,1 % (6,8 - 14,9 IC à 95 %)

**Tableau 6:** association entre le degré de malnutrition et la forme du paludisme

Paramètres		Paludisme simple	Paludisme grave	OR	IC 95%	P
Z-score PPA	<-2	67	13	2,2	1,1-4,2	0,01
	>= -2	223	97			
Z-score TPA	<-2	72	41	0,5	0,3-0,8	0,01
	>= -2	218	69			
Z-score PPT	<-2	84	16	2,3	1,3-4,3	<0,01
	>= -2	206	94			

## Discussion

L'incidence du paludisme était de 34,3%. Elle est proche des valeurs reprises dans plusieurs études hospitalières en RD Congo: 35,8% à Lwiro [1] et 38,2% à Kinshasa [12]. Dans la présente étude, le retard de croissance concerne 32,8% des cas de paludisme de l'enfant, répartis comme suit: 13,8% de manière modérée et 19,0% de manière sévère. En comparaison, la prévalence estimée du retard de croissance dans la population générale est de 41,8%, dont 20,8% dans la forme grave : chiffres proches de notre série [13]. La prévalence globale de la malnutrition aiguë (modérée à sévère) est de 32,9%, représentée essentiellement par la malnutrition sévère avec 27,5%. Le rapport MICS 2018 révèle qu'en RDC, la prévalence de la malnutrition aiguë chez les moins de 5 ans était de 6,5%, dont 2,0% sous la forme sévère [13]. Les enfants concernés par la malaria dans la présente étude étaient largement au-dessus de ces valeurs observées dans la population générale. Il est plausible de penser qu'un système immunitaire initialement faible, dû à la malnutrition, peut favoriser plus facilement le développement de l'infection palustre. Cependant, dans la littérature, cette hypothèse est controversée. Par exemple, dans une étude au Sud-Kivu, Mitangala *et al*. trouvaient que les enfants malnutris présentaient un risque plus faible de développer la malaria [2].

Dans une série gambienne, Snow *et al*. n'ont pas trouvé de corrélation entre les indices nutritionnels et la susceptibilité au paludisme, mais ont constaté que, comparés à ceux qui avaient une faible parasitémie, les enfants qui avaient une forte parasitémie avaient tendance à avoir un Z-score PPA moyen supérieur [14]. Quant à Tshikuka *et al*., au cours d'une étude de prévalence chez les enfants de 4 mois à 10 ans à Lubumbashi, ils n'ont trouvé aucune association entre la parasitémie paludéenne et les indices PPT et TPA [15]. Paradoxalement, d'autres études ont montré que la malnutrition aggravait le risque de survenue de la malaria [16-18]. Une hypothèse probable pouvant expliquer les disparités dans ces résultats est que la relation entre le paludisme et l'état nutritionnel dépendrait des nutriments présents ou non présents dans l'alimentation usuelle des enfants concernés. En effet, la malnutrition est souvent multicarentielle, et le profil clinique pourrait fortement dépendre du profil en nutriments ou micronutriments sous-jacent.

Dans notre série, une association systématiquement significative a été observée entre l'insuffisance pondérale modérée à sévère (Z-score <-2 ET), le retard de croissance modéré à sévère (Z-score <-2 ET), la malnutrition aiguë modérée à sévère (Z-score <-2 ET) et le paludisme simple plutôt qu'avec le paludisme grave.

Le surpoids a été rencontré chez 13,9% d'enfants; il était sévère chez 8,5% d'enfants. Les chiffres dans la population générale des enfants de moins de 5 ans révèlent une prévalence de 3,8%, dont 0,9% sous la forme sévère [13]. Étant donné que notre échantillon a été sélectionné essentiellement en milieu urbain, cela peut expliquer cette plus grande prévalence de la surcharge pondérale. En effet, il est établi que le risque d'obésité est accru en milieu urbain, notamment dans les classes sociales aisées, du fait de la présence de certains facteurs favorisants comme la motorisation des déplacements, la sédentarité des enfants (jeux vidéo, heures passées à suivre la télévision…) et la disponibilité accrue de la nourriture [19,20]. Une étude a montré que la comorbidité obésité et diabète constituait un facteur de risque de paludisme grave à Plasmodium falciparum, du fait essentiellement de la défaillance générale du système immunitaire [21]. Il reste à déterminer des liens éventuels chez l'enfant entre l'obésité en elle-même et la malaria.

## Conclusion

Il ressort une fréquence relativement élevée des cas d'insuffisance pondérale, d'émaciation et de retard de croissance, souvent plus importante que celle documentée dans la population générale. Étant donné qu'il s'agit d'une étude transversale, malgré les observations faites sur l'association entre l'état nutritionnel et la morbidité liée au paludisme, notre étude ne peut fournir une preuve de causalité de cette relation. Des études supplémentaires permettant de prouver le lien de cause à effet sont nécessaires. Et notamment des études évaluant l'implication des nutriments dans l'expression clinique du paludisme chez l'enfant.

### Etat des connaissances sur le sujet

- La malnutrition, aiguë et chronique, constitue un problème de santé majeur chez les enfants de moins de 5 ans vivant dans le Haut-Katanga (RD Congo);
- Le paludisme est l'une des premières causes de mortalité chez les enfants de moins de 5 ans.

### Contribution de notre étude à la connaissance

- Cette étude se penche sur le lien entre l'état nutritionnel et le paludisme chez l'enfant, lien peu exploité dans le contexte de notre milieu d'étude;
- L'étude soulève l'hypothèse qu'une amélioration de l'état nutritionnel pourrait impacter la survenue ou l'évolution clinique du paludisme.
